# The *MKK2a* Gene Involved in the MAPK Signaling Cascades Enhances *Populus* Salt Tolerance

**DOI:** 10.3390/ijms231710185

**Published:** 2022-09-05

**Authors:** Jiali Wang, Zimou Sun, Caihui Chen, Meng Xu

**Affiliations:** Co-Innovation Center for Sustainable Forestry in Southern China, Key Laboratory of Forest Genetics and Biotechnology, Ministry of Education, Nanjing Forestry University, Nanjing 210037, China

**Keywords:** MAPK signaling cascades, *PeMKK2a* gene, salt stress, overexpression, *Populus*

## Abstract

Mitogen-activated protein kinase (MAPK) cascades are highly conserved signal transduction modules, which transmit environmental signals in plant cells through stepwise phosphorylation and play indispensable roles in a wide range of physiological and biochemical processes. Here, we isolated and characterized a gene encoding MKK2 protein from poplar through the rapid amplification of cDNA ends (RACE). The full-length *PeMKK2a* gene was 1571 bp, including a 1068 bp open reading frame (ORF) encoding 355 amino acids, and the putative PeMKK2a protein belongs to the PKc_like (protein kinase domain) family (70–336 amino acids) in the PKc_MAPKK_plant subfamily and contains 62 sites of possible phosphorylation and two conserved domains, DLK and S/T-xxxxx-S/T. Detailed information about its gene structure, sequence similarities, subcellular localization, and transcript profiles under salt-stress conditions was revealed. Transgenic poplar lines overexpressing *PeMKK2a* exhibited higher activities of superoxide dismutase (SOD), catalase (CAT), and peroxidase (POD) than non-transgenic poplar under salt stress conditions. These results will provide insight into the roles of MAPK signaling cascades in poplar response to salt stress.

## 1. Introduction

As one of the major abiotic stresses, soil salinization is an increasingly serious issue that hampers plant growth and development mainly due to osmotic stress and ion toxicity. Under salt stress, excessive sodium (Na^+^) and chloride (Cl^−^) ions are taken up and accumulated by the plant root system, disrupting water homeostasis and accumulation of reactive oxygen species within plants [1,2,3]. To counteract the detrimental effects of salt-induced stress, plants have evolved diverse adaptive mechanisms [4,5]. Some important genes and regulatory pathways related to salt stress have been well-deciphered in herbaceous model plants. Compared with herbaceous plants, perennial trees have more unique and complex developmental patterns and stress adaptation mechanisms. The economically and ecologically important genus *Populus* has been adopted as an important model for basic biological research of forest trees. Soil salinization is also the main obstacle that affects the survival rate of poplar afforestation and the high yield of plantation. A better understanding of poplar salt-stress response mechanisms is crucial for breeding salt-tolerant varieties. Transient activation of the mitogen-activated protein kinase (MAPK) signaling cascades, which plays a necessary role in regulating the active adaptation of organisms to the environment, is one of the early triggered conservative defense responses [6].

MAPK signaling cascades consist of mitogen-activated protein kinase kinase kinase (MAPKKK/MAP3K/MEKK), mitogen-activated protein kinase kinase (MAPKK/MAP2K/MKK), and mitogen-activated protein kinase (MAPK/MPK) [7]. Their arrangement and successive protein phosphorylation/dephosphorylation reactions constitute the signal transduction mode of MAPK transmission chain [8,9]. MAPKKKs are located upstream of the signaling cascades, phosphorylating and activating MAPKKs on the S/T-xxxxx-S/T motif [10], and MAPKKs subsequently transmit external signals to the inside of cells through the TXY motif of the double-phosphorylated MAPKs [11,12]. Activated MAPKs can phosphorylate multiple transcription factors and other signaling pathway components [13] and participate in plant cell differentiation and development [14,15,16,17], maturation, hormone signal transduction [18], and immune processes [19,20,21]. They simultaneously act in response to all kinds of biotic and abiotic stresses [22,23].

There are 20 MAPKs, 10 MAPKKs, and 60 MAPKKKs in *Arabidopsis thaliana* [24]. *Populus trichocarpa* contains 21 MAPKs and 11 MAPKKs [25]. *Zea mays* has 19 MAPKs and 6 MAPKK members [26], whereas there are 16 MAPKs and 8 MAPKKs members in *Oryza sativa* [27]. Much knowledge has been gained about the MAPK signaling cascades in plants through analysis of the activity of endogenous and overexpressed MAP enzymes in response to different stimuli [28,29]. The 10 MAPKKs in *A. thaliana* can be divided into four groups, and each protein performs different functions in different signal transduction pathways [30]. Among these MAPKKs, MKK1, MKK2, and MKK6 are in group A. AtMKK1 is activated, and AtMPK4 phosphorylated when flg22 is used to deal with cells, causing a series of physiological and biochemical reactions to resist the invasion of pathogenic bacteria [31]. The OsMAPKKK63-OsMKK1-OsMPK4 can mediate salt signaling [32]. Unlike *O. sativa*, in *A. thaliana*, the MEKK1-MKK2-MPK4/6 cascades pathway is activated in response to salt to improve salt-stress resistance [33]. The LeMKK2-LeMPK2/LeMPK3 induced rapid HR-like cell death in *Lycopersicon esculentum* [34]. In *A. thaliana*, the stress resistance of mek1 and mek2 single mutants and the activity of MPK4 were not difference compared with those of the wild-type plants. However, in mek1/mek2 double mutants, MPK4-dependent disease-resistance signal transduction pathway was significantly affected [35]. These results implied that MKK1 and MKK2 have functional redundancy in term of mediating defense-related signal transduction [35,36]. AtMEKK1, AtMKK1/AtMKK2, and AtMPK4 function together in a mitogen-activated protein kinase cascade to regulate innate immunity in plants [37]. HINKEL kinesin, ANP MAPKKKs, and MKK6/ANQ MAPKK, which phosphorylate and activate MPK4 MAPK, constitute a pathway that is required for cytokinesis in *A. thaliana*, and MPK11 is also involved in cytokinesis [14,38]. In group B, there is only one member, MKK3. The signaling pathway formed is abscisic acid (ABA)-dependent AtMKKK18-AtMKK3-AtMPK1/2/7, which regulates leaf senescence and drought-stress resistance [39,40]. In *O. sativa*, involvement of the OsMKK3-OsMPK7-OsWRKY30 signaling pathway in response to biotic stresses [41] and the MKKK62-MKK3-MPK7/MPK14 system controlled seed dormancy by regulating the transcription of *OsMFT* [42]. Group C consists of two members, MKK4 and MKK5. When the receptor kinase FLS2 is stimulated by external pathogens, it phosphorylates AtMEKK1, which transmits external signals to the inside of cells through the AtMEKK1-AtMKK4/AtMKK5-AtMPK3/AtMPK6 signal transduction pathway, ultimately regulating the resistance of plants to pathogens and bacteria through WRKY22/29 transcription factor [14,19]. MKK7, MKK8, MKK9, and MKK10 belong to Group D. MKK7 is an inhibitor of auxin polar transport. AtMKK7-AtMPK3 is mainly involved in regulating leaf morphogenesis, and the AtMKK7-AtMPK6 level pathway is involved in regulating stem branching, geotropism of cotyledon hypocotyls, elongation of single fibers, and formation of lateral roots [43]. At present, there has been no research on the function of MKK8. The AtMKK9-AtMPK3/6 pathway regulates the activity of downstream WRKY75, affects the expression related to acidic salt ion absorption, promotes the absorption and accumulation of phosphates, and inhibits anthocyanin biosynthesis by affecting the expression of anthocyanin synthesis-related genes [28,44]. The AtMKK10-AtMPK6 pathway can be activated by red light through rapid phosphorylation and inactivation of phytochrome interacting factors, promote leaf development, and regulate plant morphogenesis in response to light [45]. ZmMKK10 and ZmMPK3/ZmMPK7 may function in the MAPK cascade and are regulating the ethylene-dependent cell death process [46]. Using a reverse genetic approach, it was found that OsMPKK10-2-OsMPK3 (OsMPK5) cascade positively to regulate drought tolerance [47].

Although there have been more studies on MAPK signaling in different herbaceous organisms in recent years, little is known on the involvement of MAPK signaling cascades in poplar response to abiotic stress [48,49,50]. In this study, a gene encoding MKK2 protein was isolated from poplar through the rapid amplification of cDNA ends (RACE); thereby, detailed information about its gene structure, sequence similarities, subcellular localization and transcript profiles under salt-stress conditions was revealed. Transgenic poplar lines overexpressing *PeMKK2a* were generated using an *Agrobacterium*-mediated leaf disk transformation, and their phenotypic characteristics under salt stress were assessed. These results will provide insight into the roles of MAPK signaling cascades in poplar response to salt stress.

## 2. Results

### 2.1. Cloning and Sequence Analysis of the PeMKK2a

**Figure 1:**
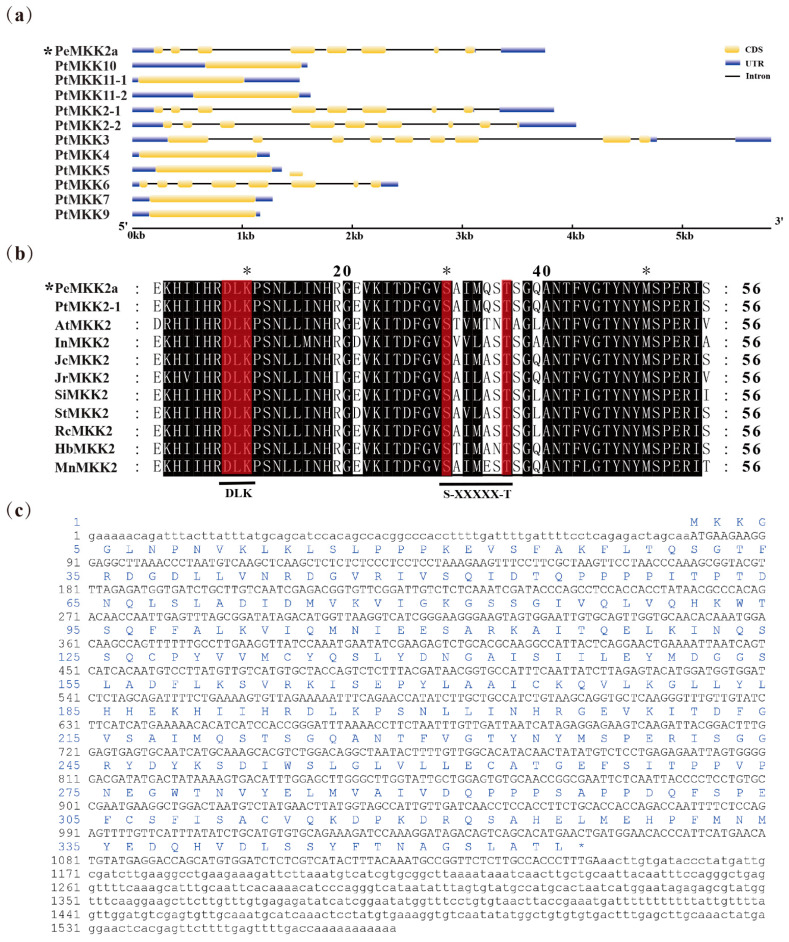
(**a**) Exon and intron composition of *PeMKK2a* and *PtMKKs*. (The * represents the *MKK2* gene cloned from “Nanlin 895”). (**b**) Multiple alignment of amino acid sequences of MKK2 proteins in different plant species. The black background indicates conserved regions, and the red background indicates specific conserved structural domains. (Here, only part of the sequence is shown.) (**c**) Nucleotide and deduced amino acid sequences of *PeMKK2a*.

The full-length 1571 bp cDNA sequence of *PeMKK2a* was successfully isolated and identified by the rapid-amplification of cDNA ends (RACE) method, yielding an open reading frame (ORF) of 1068 bp and flanked by 80 bp of 5′-untranslated region (UTR) and 423 bp of 3′-UTR. The exon-intron structure of the *PeMKK2a* gene was obtained by comparing cDNA and genomic sequences, which has eight exons with lengths of 83 bp, 85 bp, 135 bp, 225 bp, 183 bp, 224 bp, 46 bp, and 84 bp (Figure 1a). Next, the structure of the other *PtMKK* genes was also examined. *PtMKK* genes have between one and nine exons, with *PtMKK4*, *PtMKK5*, *PtMKK7*, *PtMKK9*, *PtMKK10*, *PtMKK11-1*, and *PtMKK11-2* having longer single exons. There are eight exons in *PtMKK2-1*, *PtMKK2-2*, and *PtMKK6*. Only *PtMKK3* has nine exons (Figure 1a).

The *PeMKK2a* gene encodes 355 amino acids (Figure 1c), of which leucine (Leu) and serine (Ser) are the most abundant, accounting for 9.3% of the total (Table S1). The primary structure of the PeMKK2a protein was predicted by ExPASy ProtParam. Its relative molecular weight (MW) was 39.51 kDa, the theoretical isoelectric point (PI) was 6.00, and the average hydrophilicity coefficient was −0.197, indicating that the protein has strong hydrophilicity. Using SOPMA software, we determined the secondary structure of PeMKK2a protein. The contents of random coil structures and α- helices were higher, and accounted for 38.59% and 37.46%, respectively. SWISS-MODEL software was used to obtain the tertiary structure of the PeMKK2a protein (Figure S1). The PeMKK2a protein may facilitate signaling by providing an interactive platform for components of the MAPK signaling cascades pathway. The online software program NetPhos 3.1 predicted that PeMKK2a protein contains 62 possible phosphorylation sites (Figure S2), and Ser residues constitute the largest proportion of phosphorylation sites with a total of 33.

**Figure 2:**
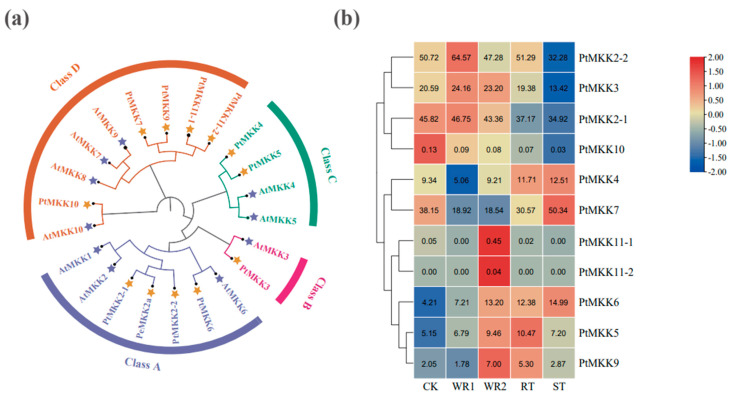
(**a**) Phylogenetic relationships of *A. thaliana* and *P. trichocarpa* MKK proteins. (**b**) Hierarchical clustering of the expression profiles of 10 *MAPKK* gene family members for different development periods and tissues in *P. trichocarpa* (CK, stem segment; WR1, one−week−old roots; WR2, two−week−old roots; RT, root tips; ST, stem tips).

The PeMKK2a protein belongs to the PKc_like (protein kinase domain) family (70–336 amino acids) in the PKc_MAPKK_plant subfamily. NCBI BLASTp search for sequences with high homology to PeMKK2a protein in different species. In all the homologous sequences, the similarity in sequence identity with sequences in woody plant species such as *P. trichocarpa*, *Hevea brasiliensis*, and *Morus notabilis* was more than 80%, but the sequence homology with those of herbaceous and crop species is relatively low. Multiple sequence alignment displayed that the MKK2 protein contained two conserved domains, DLK and S/T-xxxxx-S/T (Figure 1b), unlike the single conserved sequence S/T-xxx-S/T in mammals. Using Figtree software, we constructed a phylogenetic tree of the MKK protein family, in which we compared the isolated PeMKK2a proteins and their functionally characterized homologues from *A. thaliana* and *P. trichocarpa* at the sequence level. Among the 11 identified PtMKK proteins, the conservation within the family was low. In contrast, the proteins are highly homologous to the corresponding MKK protein in *A. thaliana*. At the same time, the PtMKK family is divided into four subgroups according to the classification of *A. thaliana*: subgroup A, subgroup B, subgroup C, and subgroup D (Figure 2a). PeMKK2a, PtMKK2-1, PtMKK2-2, and PtMKK6 belong to subgroup A. Subgroup B has only one member: PtMKK3. Group C consists of two members: PtMKK4 and PtMKK5. PtMKK7, PtMKK9, PtMKK10, PtMKK11-1, and PtMKK11-2 belong to subgroup D.

### 2.2. Expression Patterns of PtMKK Genes in Different Tissues

A transcriptome deep sequence dataset from published studies was downloaded [51,52] to characterize the expression profile of *PtMKK* genes in different tissues: one-week-old roots, two-week-old roots, root tips, and stem tips. The homologous genes *MKK2-1* and *MKK2-2* were highly expressed in all tissues and the most active in the one-week-old roots. Similarly, the expression of *MKK10*, *MKK11-1*, and *MKK11-2* in the different tissues was very low or even nonexistent. In addition, some genes, such as *MKK4*, *MKK6*, and *MKK7*, were more highly expressed in the stem tips. *MKK9* was relatively active in the two-week-old roots (Figure 2b). In addition, the expression levels of *PtMKK2-1*, *PtMKK2-2*, *PtMKK3*, *PtMKK7*, and *PtMKK10* genes decreased with root development, whereas the *PtMKK4*, *PtMKK5*, *PtMKK6*, *PtMKK9*, *PtMKK11-1*, and *PtMKK11-2* genes increased (Table S2).

### 2.3. Expression Analysis of the PeMKK2a Gene in Response to Salt Stress

**Figure 3:**
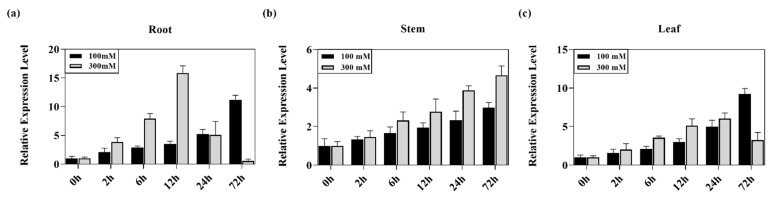
Expression pattern of *PeMKK2a* under salt stress. “Nanlin895” were exposed to salt stress at 100 mM and 300 mM for 0 h, 2 h, 6 h, 12 h, 24 h, and 72 h. Samples were taken for quantitative analysis. (**a**) Relative expression level in root. (**b**) Relative expression level in stem. (**c**) Relative expression level in leaf.

The *MKK2* gene was proven to be involved in the salt-stress signal transduction pathway in *A. thaliana* and *Musa nana* [33,53]. To fully understand whether the same function exists in poplar, the expression pattern of the *PeMKK2a* gene under 100 mM and 300 mM NaCl salt stress for 0 h, 2 h, 6 h, 12 h, 24 h, and 72 h was analyzed via quantitative real-time PCR (qRT-PCR) technology. In the roots, stems, and leaves under the salt stress of 100 mM, the expression of *PeMKK2a* showed an upwards trend over time (Figure 3a–c) (Table S3). Interestingly, the expression of the *PeMKK2a* gene peaked at 12 h when the roots were exposed to 300 mM NaCl stress, and this expression level was approximately 16 times that of the control (CK) (Figure 3a). However, there were some differences between the leaves and stems. In the leaves, the expression reached a maximum at 24 h (Figure 3c), and the expression in the stems was continuously upregulated within 72 h (Table S3), which was similar to the relative expression of the *MKK2* gene in *A. thaliana* under mild salt stress for 72 h [33]. It is hypothesized that the *PeMKK2a* gene is associated with the response to salt stress and may have functions similar with those involving the salt-stress response mechanism of *A. thaliana*.

### 2.4. Subcellular Localization of the PeMKK2a Protein

**Figure 4:**
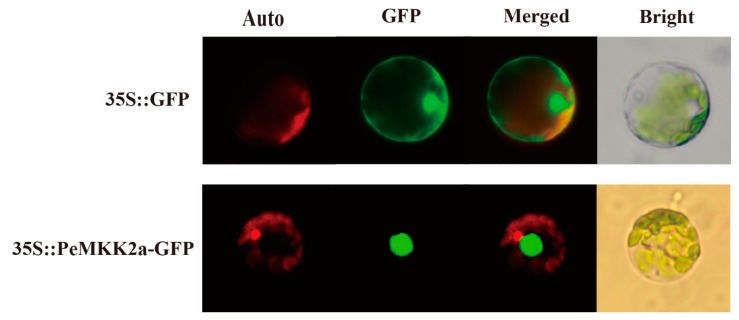
Subcellular localization of the PeMKK2a protein in poplar protoplast. Chlorophyll autofluorescence (auto), green fluorescence protein (GFP), merged and bright images are shown. (Scale bar 10 μm. The 35::GFP fusion was used as a positive protein control).

A GFP fusion vector (35S::PeMKK2a GFP) under the control of the 35S *CaMV* promoter was constructed and transformed into poplar protoplasts. Through microscopy observations, the green fluorescence expressed by the 35S::GFP control vector was distributed throughout nearly the whole cell, and the green fluorescence signal could be observed in the nucleus, cytoplasm, and cell membrane (Figure 4). On the contrary, the 35S::PeMKK2a-GFP fusion protein was detected mainly in the nucleus, consistent with those in *A. thaliana*, *P. trichocarpa*, and *Nicotiana tabacum* [54]. Thus, the *PeMKK2a* gene may have a transcriptional regulatory function in poplar growth and development.

### 2.5. Generation of PeMKK2a-Overexpressing Transgenic Lines

**Figure 5:**
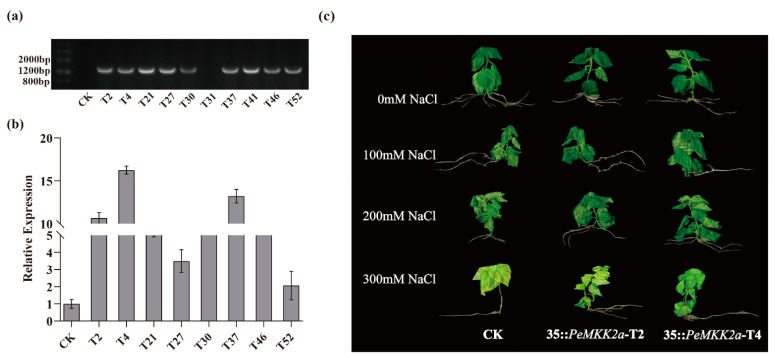
(**a**) Genomic DNA-level detection. (**b**) The expression level of *PeMKK2a* in eight transgenic lines and the CK, according to qRT-PCR (CK, non-transgenic poplar; T2, T4, T21, T27, T30, T37, T46, and T52, transgenic lines). (**c**) Phenotypic observations of transgenic plants under salt stress. (Unstressed poplar plants constituted the positive control. All the treatments included three biological replicates).

Ten transgenic lines were randomly selected for DNA content determination, and all the lines except T31 presented bands with the same size as expected (Figure 5a). The expression levels of the *PeMKK2a* gene in transgenic lines were all higher than those in the non-transgenic lines; the expression level in T4 was the highest, followed by that in T37, and the level in T52 was the lowest among these transgenic lines (Figure 5b). Clearly, the 35S::*PeMKK2a* vector was transformed into *P. davidiana × P. bolleana* and was successfully expressed, and the expression of the *PeMKK2a* genes differed greatly among the various transgenic lines, which may be caused by insertion effects at the various sites of exogenous genes in the *P. davidiana × P. bolleana* genome. The varied expression of the *PeMKK2a* gene in these lines may result differences in the resistance lines.

### 2.6. Overexpression of PeMKK2a Enhances Salt Tolerance of Poplar

T2 and T4 transgenic lines displaying high transgene expression and non-transgenic plants were exposed to 0 mM, 100 mM, 200 mM, and 300 mM salt stress to detect whether *PeMKK2a* improves salt tolerance. The growth of non-transgenic plants was inhibited after salt treatment, and under high-salt-concentration stress, the roots and stems turned black, and the leaves shriveled, turned yellow, and withered. Conversely, the T2 line had only yellow leaves, but its growth was not significantly affected. Compared with the T2 line, the T4 line was less affected (Figure 5c). The growth status of the different lines showed some slight differences, indicating that the expression of *PeMKK2a* in the transgenic plants had a certain impact on the salt tolerance of the plants, and overexpression of *PeMKK2a* improved the salt tolerance of the plants.

### 2.7. Overexpression of PeMKK2a Enhanced Antioxidant Capacity of Poplar

**Figure 6:**
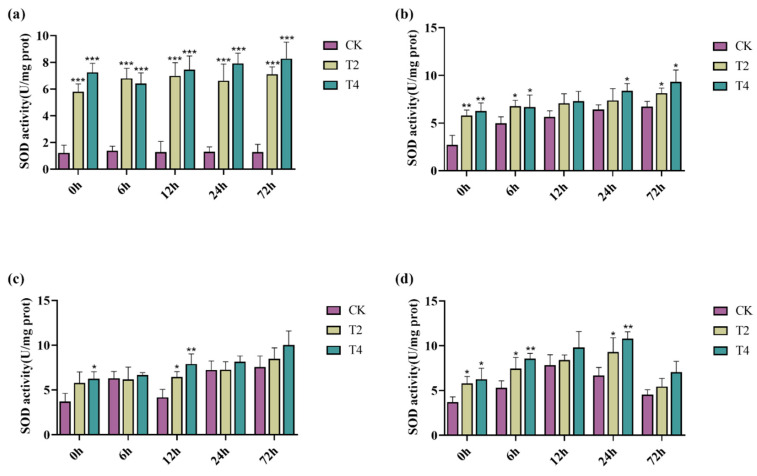
SOD activity of transgenic and non-transgenic poplar plants subjected to different NaCl concentrations. Note: (**a**) without NaCl; (**b**) 100 mM NaCl; (**c**) 200 mM NaCl; and (**d**) 300 mM NaCl (The “*” above the histogram indicates significance. “*” indicates *p* < 0.05, “**” indicates *p* < 0.01, and “***” indicates *p* < 0.001).

**Figure 7:**
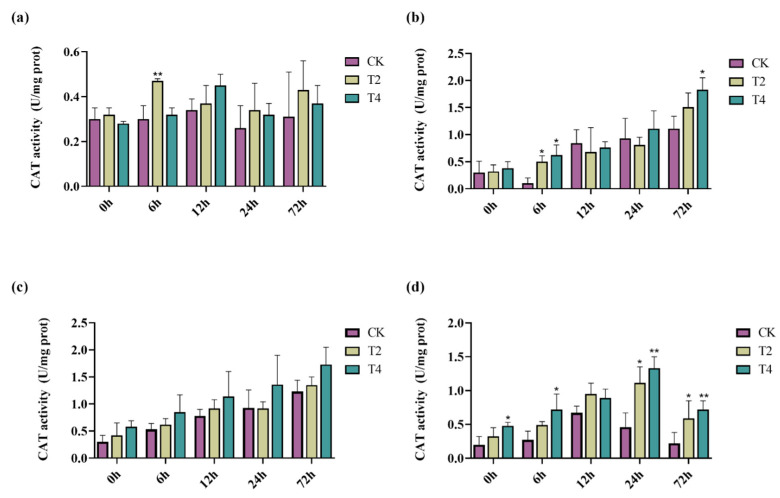
CAT activity of transgenic and non-transgenic poplar plants subjected to different NaCl concentrations. Note: (**a**) without NaCl; (**b**) 100 mM NaCl; (**c**) 200 mM NaCl; and (**d**) 300 mM NaCl (The “*” above the histogram indicates significance. “*” indicates *p* < 0.05, “**” indicates *p* < 0.01).

**Figure 8:**
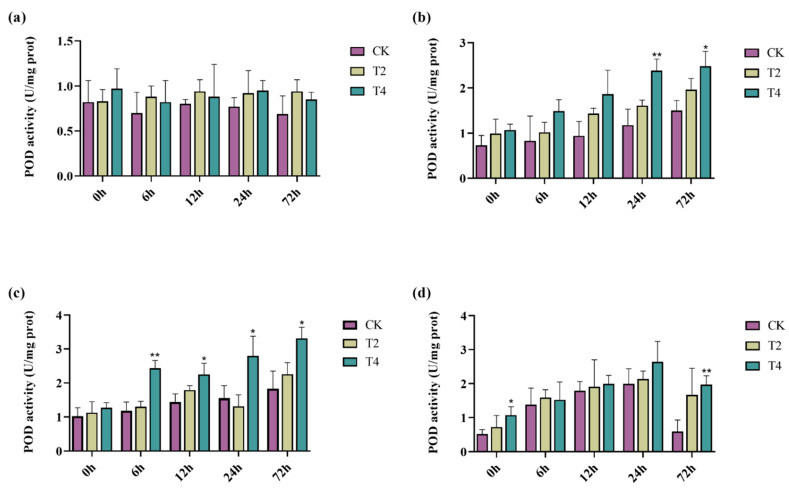
POD activity of transgenic and non-transgenic poplar plants subjected to different NaCl concentrations. Note: (**a**) without NaCl; (**b**) 100 mM NaCl; (**c**) 200 mM NaCl; and (**d**) 300 mM NaCl (The “*” above the histogram indicates significance. “*” indicates *p* < 0.05, “**” indicates *p* < 0.01).

When plants are under salt stress, they can synthesize various enzymes to reduce cellular damage caused by salt stress, such as superoxide dismutase (SOD), catalase (CAT), and peroxidase (POD) [55]. Here, we selected non-transgenic lines and transgenic lines T2 and T4 to determine the effects of the *PeMKK2a* gene on the antioxidant activity of the plants under salt stress. The antioxidant enzyme activities of the CK and transgenic lines (T2 and T4) fluctuated to a certain extent in the media without NaCl (Figure 6, Figure 7 and Figure 8). It is speculated that some subtle damage occurred when the plants were transferred to the new media, and the plants gradually overcame the effects of this damage during the growth process. Under salt stress at all concentrations, only the CAT activity of CK plants at 100 mM salt was higher than that of the transgenic plants at both 12 and 24 h, and the enzyme activity of the transgenic plants was higher than that of the CK plants under other concentrations of salt stress. Moreover, overexpression of the *PeMKK2a* gene can improve the antioxidant capacity of poplar under salt stress, and this gene may participate in the salt-stress response.

## 3. Discussion

MAPK signaling cascades are indispensable pathways in plant growth and development and in response to stress by gradually phosphorylating intracellular environmental signals and mediating the expression and regulation of functional genes. Here, 11 MKK of *P. trichocarpa* were identified according to sequences of 10 MKK *A. thaliana*. Homologous sequences of *AtMKK8* are not present in poplar, which may be a precursor of the *AtMKK7/8/9* and *PtMKK7/9/11* evolutionary branches caused by ancestral gene duplication events after monocotyledonous and dicotyledonous plant differentiation. Thereafter, *PtMKK11-1*, *PtMKK11-2*, and *AtMKK8* may drift to a nonfunctional state [56]. To date, there have been no studies on the related functions of the *MKK8* gene in *A. thaliana* and *P. trichocarpa*. Initially, in the study of the 10 MKK proteins sequences in *A. thaliana*, it was considered that *AtMKK10* lacks a correct target for constructing the activation loop and has no actual function. In 2018, it was proven that *MKK10* in *A. thaliana* is a kinase that controls leaf opening by regulating downstream MPK6 [45]. It is possible that only 9 out all 10 *A. thaliana* members have functions. The transcriptomic data showed that *MKK10*, *MKK11-1*, and *MKK11-2* were expressed at low or no levels in various tissues, which may be because only 8 of the 11 *MKK* members of poplar may have functions.

Under higher concentrations of salt-stress treatments, the CK line samples were inhibited in growth, wilted, or even died, while transgenic lines suffered less damage (Figure 5c). Under normal growth conditions, we found that the transgenic lines had the same phenotype as CK (Figure S3), a result that confirms the expression of the *PeMKK2a* gene has an effect on plant growth under salt stress.

In previous studies, MKK2 was found to be specifically activated by cold salt as well as stress-induced MEKK1 in *A. thaliana* protoplasts, and the yeast two-hybrid technique and in vitro protein kinase assays revealed that MKK2 targets MPK4 and MPK6, respectively. AtMPK4 and AtMPK6 are constitutively activated in AtMKK2-overexpressing transgenic plants with enhanced salt tolerance in AtMKK2-deficient mutants, and the activation of AtMPK4 and AtMPK6 was blocked and exhibited hyper-salinity sensitivity [33,57]. These studies suggest that the regulation of MAPKK and MAPKs under salt stress can promote MAPK cascade responses and improve salt tolerance in plants. Due to experimental time limitations, we did not verify whether MPK4 and MPK6 were phosphorylated by MKK2 in response to salt stress by protein kinase assays. However, we selected T2, T4, and T37 transgenic lines with relatively high expression and T27 and T52 transgenic lines with relatively low expression to analyze the expression levels of *MPK4* and *MPK6*. The results showed that the expression levels of the *MPK4* and *MPK6* genes were higher than those of the CK line (Figure S4). Although the up- and down-regulation relationships were different in the same lines, there was indeed a certain positive correlation with the expression level of the *PeMKK2a* gene among different transgenic lines. It is believed that these two genes may have a certain regulatory relationship with *PeMKK2a* in poplar, but the regulatory relationship and pathway need further experimental verification. Taken together, these results lay a foundation to study the downstream genes involved in *MKK2* phosphorylation in the MAPK signaling cascades in poplar.

## 4. Materials and Methods

### 4.1. Plant Materials, Growth Conditions, and Stress Treatments

The sampled plantlets of the elite clone (*P. deltoides × P. euramericana* cv. “Nanlin895”) in this study were cultivated on Murashige and Skoog (MS) medium (pH 5.8) containing 3% (*w*/*v*) sucrose and 0.2% (*w*/*v*) Gelrite in a growth chamber (SANYO, Tokyo, Japan) at 25/18 °C (day/night), daily photoperiod of 16/8 h (light/dark, illumination of 50 µmol·m^−2^s^−1^), and relative humidity of 60–80%. Transformation acceptor plantlets (*P. davidiana* × *P. bolleanan*) were maintained, transformed, and regenerated as previously described.

To determine the expression of target gene in “Nanlin895” poplar and transgenic plants, various organs (roots, stems, and leaves) from 40-day-old plants were harvested and stored at −80 °C until RNA extraction. For the salt-stress treatment, 40-day-old plants were transferred from initial solid MS medium (without NaCl) to liquid MS media containing either 100 mM and 300 mM NaCl for different times: 0, 2, 6, 12, 24, and 72 h. Two treatments of plants were used for growing, as previously described. After treatment, various tissues were harvested from three clonal plants of each treatment at each time point, frozen immediately in liquid nitrogen, and then stored at –80 °C for RNA isolation.

### 4.2. Extraction of DNA and RNA, and cDNA Synthesis

Genomic DNA was extracted from “Nanlin895” poplar using the Plant Genomic DNA Kit (TIANGEN, Beijing, China) following the manufacturer’s instructions. Total RNA was isolated using RNAprep Pure Plant Plus kit (TIANGEN, Beijing, China), and the RNA was purified by RNase-Free DNase I (TIANGEN, Beijing, China). To ensure the quality of the extracted total RNA, the NanoDrop 2000 c ultraviolet–visible spectrophotometer (Thermo Fisher, Waltham, MA, USA) was used to measure the concentration and purity. Analysis of results that the concentration of the sample should not be less than 500 ng/μL and the OD260/280 value around 2.0. Additionally, the integrity was determined by 1% agarose gel electrophoresis and guaranteed clear bands on agarose gels at 18 S and 28 S, with 28 S approximately twice as bright as 18 S. Qualified RNA samples was reverse transcribed into cDNA using TaKaRa PrimeScript^TM^ RT Master Mix Kit (TaKaRa, Dalian, China).

### 4.3. Identification and Cloning of PeMKK2a Genes

The complete amino acid sequences of 10 MKKs in *A. thaliana* were used as query sequences for BLAST homology comparisons with *P. trichocarpa* protein sequences within a database (Table S4), and the top five poplar protein sequences with the highest similarity to each *A. thaliana* MKK protein sequence were selected. Oligo7 software was used to design specific amplification primers and RACE primers (Table 1). According to the operation instructions, the 3′-Full RACE Core Set with PrimeScript^TM^ RTase (Takara, Dalian, China) and the 5′-Full RACE Kit with TAP (Takara, Dalian, China) were used to amplify the full-length *PeMKK2a*. The isolated fragment was ligated to a pMD-19T vector and transformed into TOP10. Positive detection and sequencing were performed to obtain the nucleotide sequence of the target fragment. The 3′ RACE and 5′ RACE sequences were compared and spliced, and the NCBI ORFfinder (https://www.ncbi.nlm.nih.gov/orffinder/, accessed on 10 October 2021) was used to predict open reading frame (ORF). Afterward, we designed specific primers targeting both ends of the potential ORF (Table 1), conducted PCR amplification, and performed sequencing verification.

### 4.4. Sequence Analysis

The online software ExPASy ProPARma (https://web.expasy.org/protparam, accessed on 18 October 2021), SWISS-MODEL software (https://swissmodel.expasy.org/, accessed on 18 October 2021), SOPMA software (https://npsa-prabi.ibcp.fr/cgi-bin/npsa_automat.pl?page=npsa%20_sopma.html, accessed on 18 October 2021), and NetPhos 3.1 software (http://www.cbs.dtu.dk/services/NetPhos/, accessed on 20 October 2021) were used to obtain physicochemical properties of the PeMKK2a protein. The conserved domain of *PeMKK2a* was determined according to information within the NCBI Conserved Domain Database (CDD). ClustalX v2.1.1 software (https://github.com/search?q=ClustalX, accessed on 5 November 2021) was performed multiple protein sequences comparison of MKK2 protein from various plant species, and we manually adjusted these acids sequence by GeneDoc 2.7 software (https://genedoc.software.informer.com/2.7/, accessed on 22 November 2021). Figtree v1.4.4 software (http://tree.bio.ed.ac.uk/software/Figtree/, accessed on 29 November 2021), was used to construct a systematic evolutionary tree, and transcriptome data (RNA-seq) were used to analyze the expression of the *PtMKK* gene family. 

### 4.5. Expression Analysis of the PeMKK2a Gene of Poplar under Salt Stress

The expression patterns of *PeMKK2a* gene under salt stress were detected using qRT-PCR technology, performed on PowerUP^TM^ SYBR^TM^ Green Master Mix (Thermo Fisher, Waltham, MA, USA). The qRT-PCR-specific primers were designed by Beacon Designer 8 software (Table 1), and the *18S* gene was selected as the reference gene [58]. Each experiment had three technical replicates, and the relative quantitative analysis was performed using the 2^−∆∆Ct^ method [59].

### 4.6. Transient Expression Vector Construction and Transformation

The transient expression vector was constructed by Gateway technology. The BP reaction was used to ligate the coding region of the *PeMKK2a* gene without the stop codon into PCR^TM^8/GW/TOPO^TM^, after which the vector was transformed into the *Escherichia coli* competent cells, positive clones were screened, and plasmids DNA extracted (the BP reaction and LR reaction were performed according to the operation manuals of the systems used).

Using a well-established poplar leaf pulp protoplast transformation system [60], we transferred the GFP fusion vector construct (35S::PeMKK2a-GFP) into “Nanlin895” protoplasts. The transformed protoplasts were cultured in the dark at 28 °C for 16–18 h, and the GFP fluorescence signal was observed under a laser confocal microscope.

### 4.7. Overexpression Vector Construction and Transformation

The ORF sequence of *PeMKK2a* was used to design specific primers (Table 1) linked to the overexpression vector pH35GS by Gateway technology. Introduction of a vector containing the Pro35S::*PeMKK2a* gene into *Agrobacterium tumefaciens* strain EHA105 for genetic transformation of *P. davidiana × P. bolleana* was performed.

### 4.8. Transgenic Poplar Confirmation and Salt Stress Treatment

After screening for hygromycin B (Hyg) and temetine (Tim), positive identification of transformed *P. davidiana × P. bolleana* was performed to determine whether the transgene had been successfully inserted into the genome of the recipient plant, and molecular detection, including DNA–PCR detection and qRT-PCR detection, was carried out for transgenic plants of different lines and non-transgenic *P. davidiana × P. bolleana* (CK) plants. The specific amplification primers used are shown in Table 1. The transgenic *P. davidiana × P. bolleana* and CK plants were placed in semisolid rooting screening media supplemented with 0 mM, 100 mM, 200 mM, and 300 mM NaCl for 2 h, 6 h, 12 h, 24 h, and 72 h, respectively. Three samples were taken as biological duplicates and stored at −80 °C for subsequent physiological index testing after being flash frozen in liquid nitrogen. After the treated transgenic plants were removed from the media, the intact plants were scanned to compare and analyze their phenotypes.

### 4.9. Physiological Assay

We precisely weighed 0.05 g of the above transgenic plant samples and CK samples to prepare tissue homogenate and used a total protein quantitative test kit (BCA method), SOD test kit, CAT test kit, and POD test kit to detect the total protein content, SOD activity, CAT activity, and POD activity, respectively, in transgenic plant samples and the CK samples. We referred to the operating instructions of the kits for all our experimental methods.

## 5. Conclusions

In our study, we demonstrated that the *PeMKK2a* gene is a positive regulator of salt tolerance in poplar, consistent with the function of the *MKK2* gene in *A. thaliana*. This result enriches our understanding of the salt-stress signaling pathway in poplar and provides a reference for the study of salt-resistance mechanisms in other woody plants. Meanwhile, the downstream genes involved in the salt-stress pathway were not identified, which provides a guide for our future studies.

## Figures and Tables

**Table 1:** Primers used in this study.

Primer_ID	Forward PCR Primer (5′-3′)	Reverse PCR Primer (5′-3′)
*PeMKK2a_*ORF	ATGAAGAAGGGAGGCTTAAACCCTAATG	TCAAAGGGTGGCAAGAGAACC
*PeMKK2a_*3OUTER	ATCGATACCCAGCCTCCACCACC	CGCGGATCCACAGCCTACTGATGATCAGTCGATG
*PeMKK2a_*3INNER	TGGTTAAGGTCATCGGGAAGGGAAG	TGGTTAAGGTCATCGGGAAGGGAAG
*PeMKK2a_*5OUTER *PeMKK2a_*5INNER	GCACTCACTCCAAAGTCCGTAA ACAGATGGCAGCAAGATATGGTT	CGCGGATCCACAGCCTACTGATGATCAGTCGATG TGGTTAAGGTCATCGGGAAGGGAAG
*PeMKK2a_*qRT-PCR	CCCTCCTGTGCCGAATGAAGG	TGGGTGTTCCATCAGTTCATGTGC
*PeMPK4_*qRT-PCR	ACTCACGGCGGCCAATTCAT	CGCCACCATCTCGTTCGTCT
*PeMPK6_*qRT-PCR	AGGAGGAGGAGGTGGAGGGA	CAACGCCGAACAGACGATGC
*PeMKK2a*_p35Sf3	AGGAAGGTGGCTCCTACAAATGCCATC	TCAAAGGGTGGCAAGAGAACC
*18S* [55]	TCAACTTTCGATGGTAGGATAGTG	CCGTGTCAGGATTGGGTAATTT

## Supplementary Materials

The following supporting information can be downloaded at: https://www.mdpi.com/article/10.3390/ijms231710185/s1.
