# Supplementary figures and images for: The MKK2a Gene Involved in the MAPK Signaling Cascades Enhances Populus Salt Tolerance
Source: Int J Mol Sci. 2022 Sep 5;23(17):10185. doi: 10.3390/ijms231710185 (PMC9456161; doi:10.3390/ijms231710185)

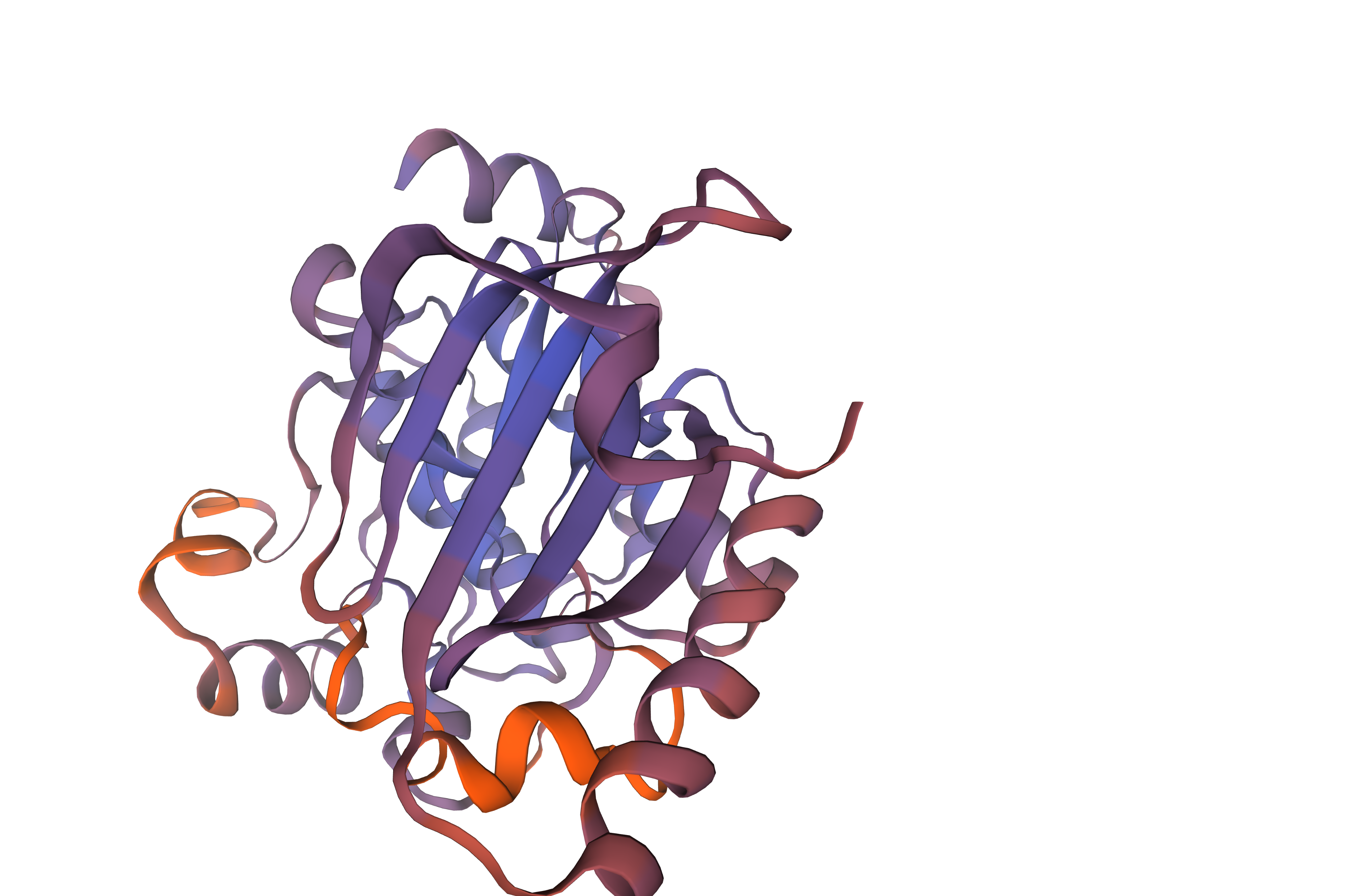

Supplement: Supplementary file 1 [file ijms-23-10185-s001.zip › Figure S1.png]

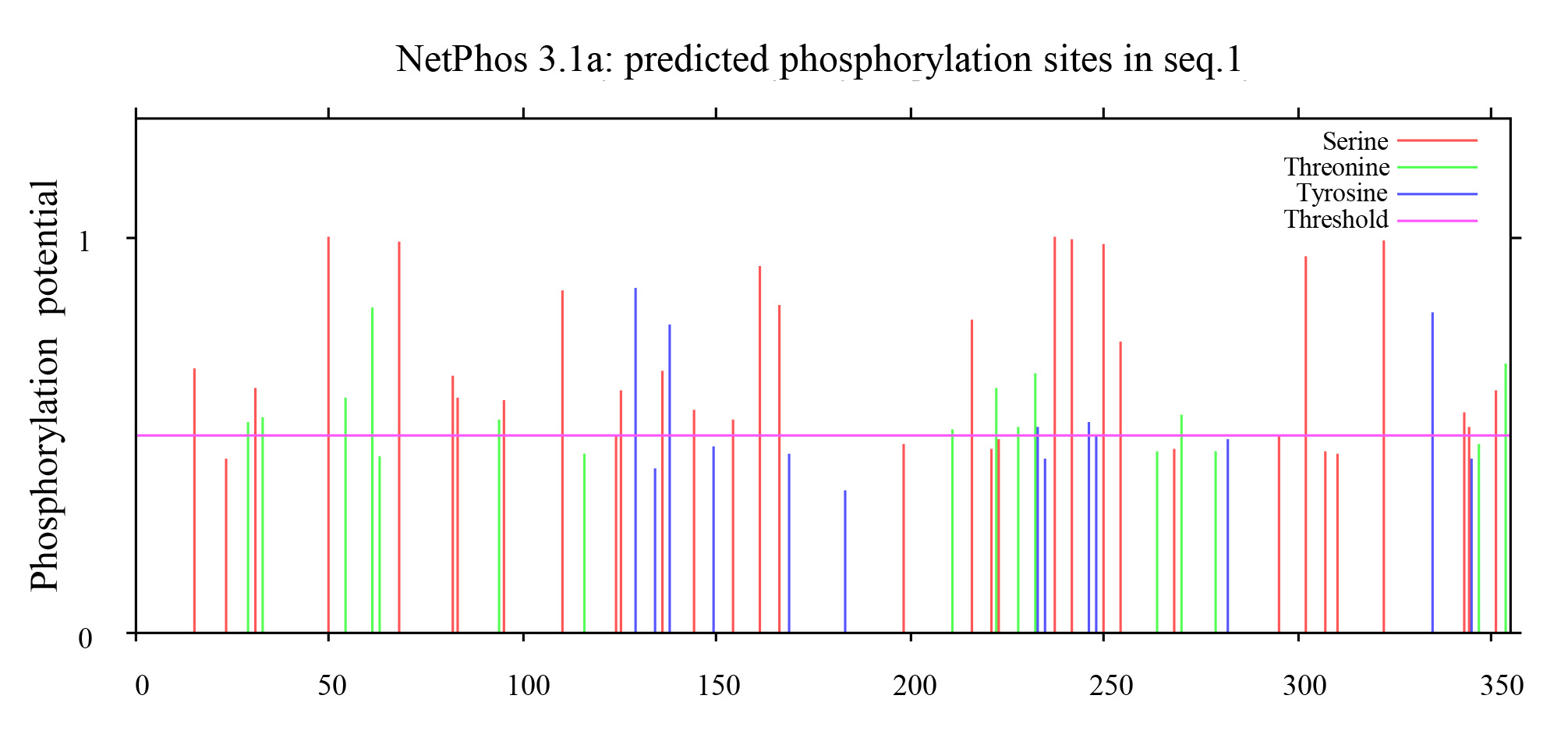

Supplement: Supplementary file 1 [file ijms-23-10185-s001.zip › Figure S2.png]

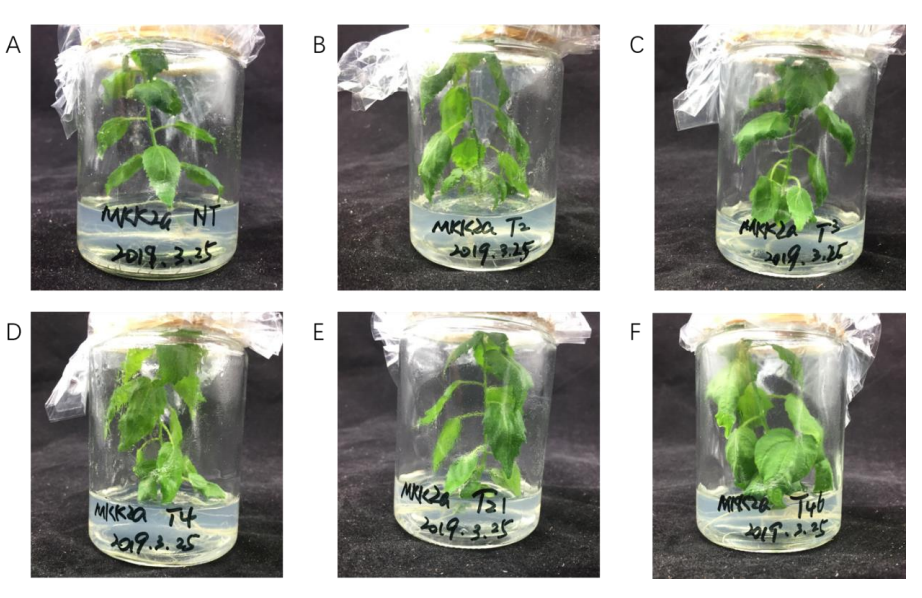

Supplement: Supplementary file 1 [file ijms-23-10185-s001.zip › Figure S3.png]

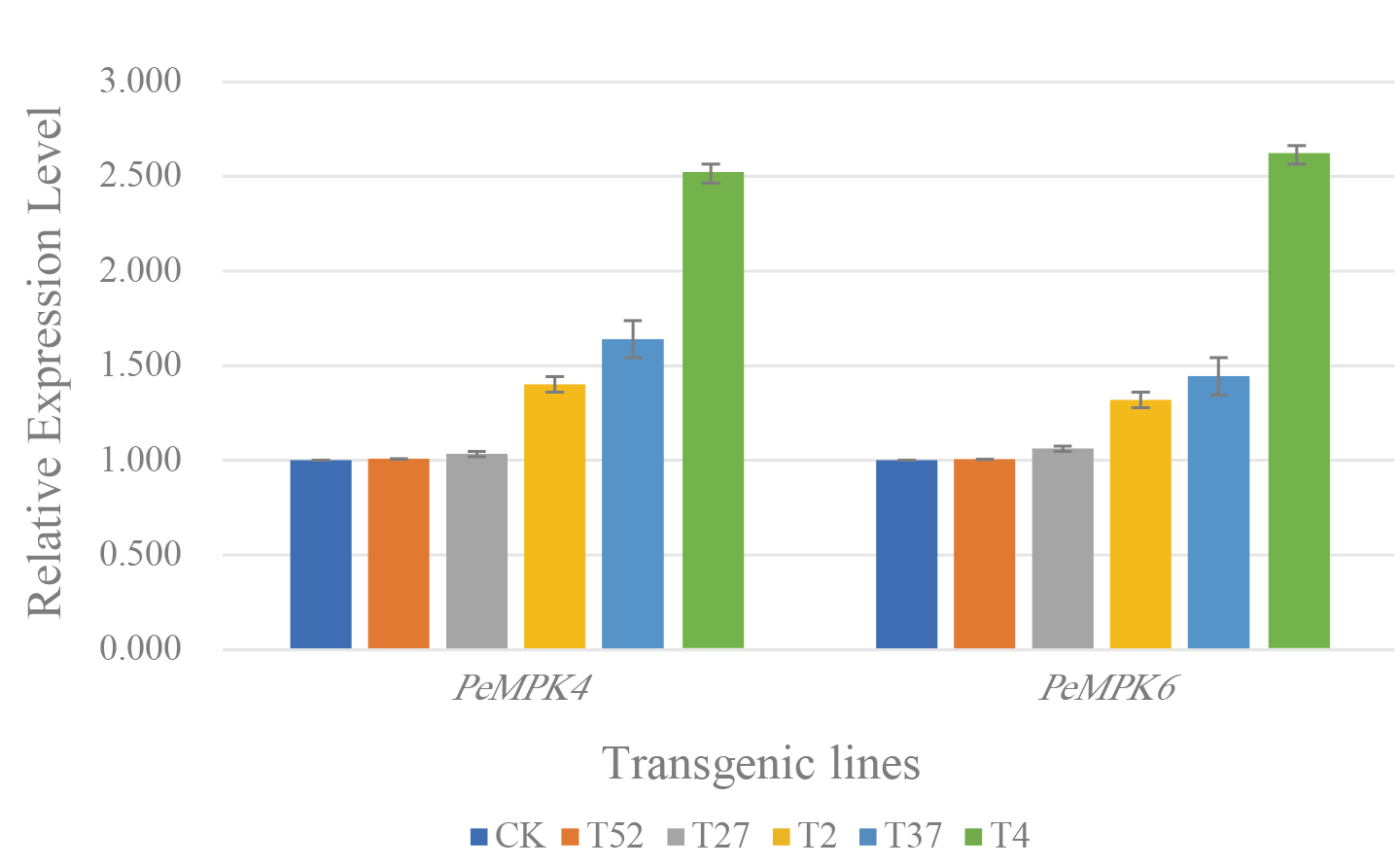

Supplement: Supplementary file 1 [file ijms-23-10185-s001.zip › Figure S4.png]
